# On Cleft Palate, and on Staphyloraphy

**Published:** 1848-04

**Authors:** William Fergusson

**Affiliations:** Professor of Surgery in King's College, London.


### ARTICLE IX.

From the Medical Times.

*On Cleft Palate, and on Staphyloraphy.* By William Fergusson, Esq., F. R. S. E., Professor of Surgery in King's College, London.

[Continued from page 192.]

Surgeons have long been familiar with the difficulty of keeping the parts quiet during, but especially after an operation, and many ingenious proposals have been made to obviate this. The most simple, and, perhaps, most reasonable was the plan of after-treatment pursued by Roux. The patient was enjoined to silence, and to abstain from any effort at deglutition; he was not permitted to swallow his saliva even. But this did not appear sufficient in all cases, and the distinguished author of the operation proposed to separate the soft palate from the hard by a transverse incision, and the plan was afterwards recommended by Mr. Bushe of New York.* This method was advised in examples where the gap was large and involved the hard palate, and it would certainly permit the anterior parts of the soft palate to be more readily brought into contact. Dieffenbach and Pancoast† have split the flaps.

The latter gentleman has described the process thus: "When the knots were prepared for tying, but before they were finally secured, Wenzel's cataract knife was passed from before backwards, through the attached side of the palate, to enable the two halves of the velum to come together in the middle line, as well as to divide the insertion of the palate muscles, so as to prevent them straining the outward edges of the palate asunder." Mr. Liston‡ has advised that, "before the ligatures are finally secured, the parts being put on the stretch, an incision should be made on each side towards the alveolar ridge, through

* An Essay on the Operations for Cleft Palate. By G. Bushe. New York, 1836.

† American Journal of Medical Science, vol. xxxii, p. 71.

‡ Operative Surgery.

the anterior surface of the velum, by which method the edges come together more easily, and the strain is taken off the threads, so that there is less risk of them making their way out by ulceration."

Dr. Mettauer of Virginia,* has proposed to increase the breadth of the two flaps, by making various incisions in the palate. One plan is, to make a series of lunated wounds through the flaps, each about half an inch in length, along the margins of the fissure, which are permitted to heal by granulation, and thereafter the ordinary operation is performed. Another plan of Dr. Mettauer's is, to make a long incision on the lower surface of the palate on each side of the fissure, at the time of doing the operation.

Dr. J. M. Warren has stated† that he had found the following course to be invariably followed by success: "The soft parts being forcibly stretched, a pair of long powerful French scissors, curved on the flat side, are carried behind the anterior pillar of the palate; its attachments to the tonsil and to the posterior pillar are to be carefully cut away, on which the anterior soft parts will at once be found to expand, and an ample flap be provided for all desirable purposes."

From the variety of plans here alluded to, it will be observed, that the difficulty of bringing the edges of the gap together, of preventing dragging on the stitches, or subsequent separation of the newly-united parts, had not been overlooked. It is singular enough, however, that none of them have had reference to the correct anatomy of the parts; indeed, scarcely an allusion has been made to this subject. Dr. Pancoast is, perhaps, the only party who has alluded in apparently precise terms to the muscles of the parts, for his incisions are intended, to use his own language, "to divide the insertion of the palate muscles." It so happens, however, that no palate muscle is inserted exactly in this direction. The line marked out runs parallel with the fibres of the palato-pharygeus; and were the le-

*American Journal of Medical Science, vol. xxi, p. 309.
†New England Quarterly Jour. of Medicine and Surgery, April, 1843.

vator-palati divided in the incision, it would only be by chance; indeed, it would be next to impossible to divide it in such a way; the knife would, in all probability, pass through the palate either on the inside or outside of the lower end of the muscle. In the quotation already given from Mr. Liston's work no allusion is made to the anatomy of the parts, and it does not appear that more was intended than to permit that relaxation which a gap on each side might be supposed to produce as regards approximation in the mesial line. In the last edition of the excellent volume alluded to, which has come out since the appearance of my paper on cleft palate, and after the author inspected my dissection, it is advised that the incision "through the anterior surface of the velum" should be made "well down by the sides of the uvula." It is stated, too, after allusion is made to my proposal, that "if the fleshy belly of the circumflexus palati could safely be reached and cut, this would, so far as I can understand, put the parts in a still more favorable condition to come together. Its tendon is certainly divided by the incision above directed, properly and effectually carried out." Doubtless it had escaped Mr. Liston's memory that my preparation proves that the circumflexus muscle has scarcely any influence on the palate—a circumstance which I have alluded to in my paper. The "fleshy belly" of this muscle may be reached with nearly as much safety and facility as that of the levator, but it seemed to me that so little good would result from its division, that I was content with the statement as to its comparatively unimportant action. An incision "through the anterior surface of the velum" would not include any portion of the circumflexus, and one "well down by the sides of the uvula" could not possibly reach the tendon of that muscle. Mr. Liston* justly observes that "the union is apt to fail under any circumstances," and, moreover, adds, in reference to my own views, "and I think that this was found to take place in the hands of the above-named professor, even after the division of the muscles as he has recommended"—a thought, the accuracy

* "Operative Surgery," 4th ed. p. 572.

of which I cannot impugn, although the reflections on this subject do not seem to me to have been either so extensive or founded on such accurate data as one might have expected, in an authority so unquestionable and so impartial: for it does not appear that Mr. Liston had remembered the two successful cases which were detailed in my paper presented to the Medico-Chirurgical Society, nor the statement* which I had subsequently placed in his hands, that I had been successful with the practice in six instances out of eight wherein I had performed the operation in the manner alluded to.

In Mettauer's plans, no allusion is made to the anatomy of the parts, some of the little wounds through the palate must implicate a few fibres of the palato-pharyngeus, but the long incision on the lower surface of the palate cannot touch any muscular fibres. In Dr. J. M. Warren's incision with the scissors, a portion of the palato-pharyngeus might possibly have been divided; but this plan, like all the others above alluded to, while intended to produce mechanical relaxation, had no special reference to the anatomy or physiology of the parts.

There are cases of cleft palate with which it would be unreasonable to meddle: the gap being so large and the soft tissues so narrow, that union could not possibly be anticipated. It has been supposed that when the two portions of the uvula are observed to touch each other during deglutition, the operation may invariably be undertaken; but the fact is, that in almost all instances these two parts touch at this particular time, however large the fissure may be, and it is better to be guided in deciding upon the propriety of an operation by the condition of the parts otherwise. In most cases where the osseous palate is open, there will be less certainty of a favorable result than if the soft velum alone were implicated. If it seems that only a small portion of the fissure in the soft parts can be closed, it will perhaps be best to leave the parts alone, and to trust for improvement entirely to an obturator or false palate, for it has sometimes been found that when there has been union

* "System of Practical Surgery," 2nd ed. p. 532.

only to a small extent, the condition has interfered with the proper adaptation of the apparatus.

The operation should seldom be undertaken until the patient has reached puberty. Much steadiness and self-command is required on his part, both during the operation and afterwards; and it is hardly to be expected that one under this age will have the fortitude to do what the surgeon expects of him. I have, in one instance, seen a youth of eleven years of age comport himself admirably during the operation; but any time between sixteen and four-and-twenty is that which should be preferred.

The mode of proceeding which I generally follow may be thus described:—The patient should be seated on a firm chair with his face to the light; the surgeon should stand a little in front, on the right side, and occasionally behind the patient. In this latter position he may see into the mouth by leaning over the face, and use his fingers with more satisfaction and facility than if he were always in front, for here he is apt to obstruct the light, and possibly fatigue his hands by holding them so long in an elevated position towards the roof of the mouth. With a knife, I make an incision, about half an inch in length, a little above the free margin on each side of the cleft, whereby the levator palati muscle is divided. The knife is sharp at the point, and also at each side, so that it may be readily passed through the mucous membrane and carried backward and forwards to enlarge the wound to the requisite extent. The point of the blade is entered above the middle part of each soft flap, where there is the greatest thickness of tissues, and, whilst it is carried deep against the levator muscle, it is moved as just directed, and not withdrawn until the power of elevating the part seems to be done away with. If, when the knife is withdrawn, there should still appear strong muscular action in an upward direction, as may be ascertained by irritating the parts, it may be used again, as possibly the whole of the muscle may not have been cut across. All this can be best done while standing at the patient's side. The edges of the fissure should now be pared; the mucous membrane on the middle part of each margin should be seized with hookbeaked forceps, and transfixed with a narrow, sharp-pointed blade which should then be run backwards and forwards so as to remove a slip of the membrane throughout the whole line of the gap. I have found it most convenient at this stage of the proceeding to stand before the patient whilst paring the left side, and behind him while cutting on the right side; but if the surgeon can hold the different instruments in each hand with equal facility he may stand as he chooses. During the time, and more especially, after these incisions are made, small pieces of sponge wrung out of iced water should be applied to clean the parts from blood and mucus; and the patient may also gargle the throat with cold water. The stitches should next be introduced thus:—A needle, set in a handle, armed with a portion of stout silk thread, three quarters of a yard long, should be passed through the soft flap about a quarter of an inch from the free margin, half an inch or less from the posterior edge of the osseous palate, from below upwards, and when the eye appears above or in the gap, the thread should be seized and drawn into the mouth with forceps; while the needle is withdrawn the end of the ligature (as yet double) should be brought out from the mouth to facilitate future steps, and also to prevent slipping. The same needle, or another like it, armed with a thread of a similar length but much thinner, should be passed in like manner through the other side of the left palate, exactly opposite the first puncture, and similar maneuvres should be repeated. By fixing this second thread to the bent end of the first, where it is hanging out of the mouth, and then withdrawing it in the course through which it has already passed, the thread intended to form the stitch will thus be brought through the opposite side of the palate, when one end of it (for it has as yet been double) can be drawn out so as to leave both ready for knotting. Two, three, or four more threads, as may seem requisite, can be introduced in a similar manner; and now all that remains to be done is to draw the edges together and fasten the thread. The foremost thread should be first tied in accordance with the ordinary mode of making the interrupted suture; and the others should then be treated in the same order in which they have been introduced. Should an additional suture seem requisite in any part of the fissure, it may now be introduced by pushing the same needle from one side to the other—for now, when the parts are more fixed by the sutures, this may readily be accomplished. Before fastening the two knots furthest back, the pared edges should be brought together to ascertain the influence of the palato-pharyngeus in dragging them asunder. If this action seems strong, or if there be difficulty in drawing the parts together, the threads should be pulled forwards, whereby the posterior pillars of the fauces will be put upon the stretch, when each should be cut about half an inch behind the uvula in an outward direction, to the extent of a quarter of an inch, and then there will be greater relaxation. Long curved scissors with blunt points, are such as I use for this part of the operation, and the same are good for cutting off the ends of the ligatures, which is the last step in the operation.

In some instances it may appear best to effect the division of the palato-pharyngeus before passing the stitches. If this be desired, the fibres can be put on the stretch by drawing the uvula forwards with the beaked forceps. It will rarely seem requisite to meddle with the palato-glossus, but if its division is thought advisable, the scissors just described will be the best instrument to use. A small horizontal wound in front of the tonsil, and about midway between the tongue and palate, will suffice.

The hookbeaked forceps, and also those for seizing the threads, should be a little longer than those in common use; and the curved needle is similar to that often employed for the strangulation of hemorrhoids, nævi, and such like growths.

I have named a stout silk ligature, as I think it preferable to any other kind. Sometimes I have used a hempen thread, but it is difficult to get the material sufficiently small and strong at the same time. I have never used the lead ligature, as recommended by Dieffenbach and others, and, from my experience of the operation, should not feel inclined to try it. The threads to be used should be well rubbed with wax, and it is highly advantageous to have them of different colors, whereby they can be more readily recognised during the proceedings.

In the ordinary operation, it has been found, on attempting to cast the common knot for the interrupted suture, that the first turn of the thread is apt to slip ere the second can be drawn. To prevent this, the points of the common forceps have been closed upon the first until the other has been brought upon it; or the surgeon's knot has been used* in expectation that the first twist of it being double, there should be less risk of slipping. Instead of a knot, Sir Philip Crampton† has passed the two ends of the thread through an aperture in a bead of soft metal, which he has squeezed close upon them at the proper distance. Mr. Brooke has, with an ingenious method, by means of glass beads, proposed to improve the style of suture here. The common knot and the surgeon's I have used most frequently, for I have always supposed that the beads might increase the after irritation. Besides, I feel satisfied that, in the operation which I perform, there is far less dragging on the threads than under ordinary circumstances, and that there is consequently less tendency to slip. But the slightest elasticity in the lateral flaps, unless indeed they be very broad, will be apt to produce a slip; and, to obviate this, I imagine that a knot of this kind will be found very serviceable. On one portion of the thread I cast a loose loop, with a single turn; the other end being then passed through it, the loop is drawn tight, and the fingers are then pushed towards the roof of the mouth and margins of fissure, as with an ordinary knot. If the loop is drawn tight there is no risk of slipping; it should hold, in fact, as if a metal bead were squeezed somewhat tightly upon the end within it. When sufficient tightness is secured, as regards the wound, a knot should be cast on the two ends of the thread, as in the common mode of fastening an interrupted suture. Professor Pancoast has advised that the knots should

* Professor Smith and Dr. J. Mason Warren.

† "Dublin Journal of Medical Science," July 1, 1843.

not be left in the mesial line, where they would be exactly over the wound, but that they should rather be kept to a side. It will be found easier to do this with the knot I have just recommended than with the common one, or more especially the surgeon's; and, as I believe it is rather an advantage to keep them off the wound (for the ends are apt to lodge in it, thereby preventing union to a certain extent, and causing irritation on the raw surfaces,) I advise you to think of this plan, which seems to me to embody the advantages of the beads, while the knots will, from their size, be less annoying to the patient than the materials alluded to.

When the operation for cleft palate is performed in the ordinary manner there is generally so much muscular spasm as to cause great difficulty in paring the edges, introducing the needles, as well as bringing and holding the cut edges together. By the plans recommended by me, these difficulties are entirely done away with, or greatly modified. The first incision is intended to take off the influence of the levator palati: if it be successful the palate seems to drop a little, and it is not so forcibly dragged upwards and outwards as under ordinary circumstances. Some movement of this kind may possibly still be present, and it may depend upon some of the fibres of the muscle having escaped the knife. The palato-pharyngeus, being still entire, will draw the margins of the fissure outwards; but when this muscle is divided there will then be no longer any action of the kind. This muscle, however, has so little influence compared with the levator, that it seems to me advisable not to divide it on all occasions until the probable amount of dragging upon the stitches has been ascertained. Even when its fibres are cut there will be some convulsive movement in the lateral flaps, for the part between the section and the attachment in front will be in some degree under the influence of muscular contraction; a shortening may take place when the parts are irritated, and this movement will be aided by the azygos uvulæ, which throughout the whole proceedings remains untouched unless when paring the edges or passing the stitches. Whatever irritability there may be, however great the spasm, there will certainly be less difficulty in passing the needles, and less opposition to the closure of the fissure, than under ordinary circumstances. After the sutures are fastened, the parts are more quiescent than with the muscles entire; indeed, in the course of a few hours (if not immediately after the operation) the roof of the mouth may be touched or tickled as you choose, and there will scarcely be any movement observed. There is one advantage in the incision which I make on the upper surface of the flaps, which is probably not the least that I claim for my own mode of operating. The wound is in the course of a few hours filled with lymph, which so thickens and stiffens the palate that any twitching of muscular fibres that may have been observable before, are no longer apparent.

However efficiently the incisions which I recommend may be executed, the operation may, nevertheless, fail. The ordinary operation for harelip, when performed even under the most favorable circumstances, will sometimes fail, and occasionally a simple incised wound in the skin will not unite. Such results are still more likely to happen in the operation for cleft palate. The causes of failure may be as obscure as they often are in other wounds, but sometimes we may see reason why it has not taken place, and it may be well to refer to some of the circumstances likely to lead to an unfavorable issue.

The grand immediate object of the operation is to obtain union by the first intention, and this may be thwarted in various ways by the surgeon himself.

Hitherto, the principal cause of failure has probably been the dragging on the stitches from the action of the muscles, and the consequent disposition for the parts to be drawn asunder; and this cause, I would fain hope, may now be in a great measure obviated by the proceedings which I recommend. There may be a deficiency of adhesive action, or there may be an excess of inflammation. In one case the gap may fly open almost as soon as the stitches are removed, in another, the process of ulceration may produce the like effect after the lapse of days. Sloughing may actually occur, and there may be defects in the performance of the operation to account for this as well as otherwise to cause failure. I believe that extensive incisions, whether on the plans recommended by Roux, Dieffenbach, Mettauer, Liston, Warren, or myself, may possibly induce defective circulation or excess of inflammation, and from either of these conditions sloughing is likely to happen. The stitches may be so numerous as to do harm, especially if drawn so tight as to impede circulation, or possibly there may be so few that the surfaces are not properly held together. The needles may be introduced too far from the margins, or not far enough, or perhaps the margins themselves may not be sufficiently pared. I imagine, too, that evil may arise from awkwardness in effecting the whole proceedings: for, if there be much manipulation, much squeezing, pinching, or poking with knives, needles, and fingers, the chances of adhesion are thereby diminished.

After the operation is finished, every care must be taken that nothing be allowed to interfere with the process of union. It is very certian that, if the patient were permitted to use the parts in the ordinary way, such as in eating, drinking, or even swallowing the saliva, they would be greatly disturbed, and adhesion might not occur. If all the muscles of the palate be left entire, as is the case in Roux's operation, the least effort at deglutition will cause considerable spasm and dragging on the stitches, and in examples where the gap is large, it is easy to perceive that much harm might result on such an occasion. Even in the proceeding which I follow, perfect quietude cannot be obtained, for the tongue below, and the constrictors behind and above, will still, during the act of swallowing, have such influence upon the palate, as greatly to disturb the healing action. It is requisite, then, to restrain the patient from such evil chances until adhesion has become so firm that it cannot be readily severed. Roux used to prohibit the use of food for eight-and-forty hours or more, and to prevent the party swallowing even his saliva. But danger may arise from too strict an adherence to this practice: patients have been known to take food at all risks and so break up the adhesions; others have become temporarily deranged; and the sudden deprivation from food has caused considerable shock to the system in many instances. Sir Philip Crampton* has not acted rigidly upon this rule, and has permitted some of his patients "boiled bread and milk, custards, soups, and jelly, twice or three times a day." In most of my cases I have given the patient gruel, soup, and wine frequently during the day, and have invariably noticed that those thus treated have recovered more rapidly than the others who have been refused all food and drink. The custom of permitting a hearty meal one hour before the operation should not be neglected; but there is great chance of its being ejected from the stomach during the proceedings, for there are few patients who do not get squeamish during the dealings with the palate. Indeed, I have remarked the chances to be greater in those who have partaken most largely of food beforehand.

A tickling cough almost invariably comes on a few hours after the operation, especially if the uvula swells much, as it often does—so that it actually drops on the root of the tongue and epiglottis, and I have found a draught containing sixty minims of the compound tincture of camphor, at bedtime, to be of great service in such cases. The bowels are usually constipated for the first two or three days, and I generally add a drop of croton oil to such a draught, with good effect. The patient may keep in bed, or sit up during the day, as he may incline; and, as a matter of course, when he is swallowing what you permit, he ought to do so with caution. On the second or third day after the operation, one, two, or more of the stitches should be removed; on the third or fourth day, if they have not already all been taken away, this should be done; they are more likely to do harm than good, if permitted to remain beyond this time. But the surgeon must use his discretion on this point, as indeed is necessary as regards all the after treatment, for I do not think it reasonable to give out rules which shall answer all cases. The mucus which accumulates about the roof of the mouth, particularly in those cases where there is an aperture left in the hard palate, must be taken carefully away, once or twice a day, with forceps, and it answers well to dry

* "Dublin Journal of Medical Science," July 1, 1843, vol. xxii.

the parts with a bit of soft sponge, which has been previously wrung out of a weak solution of nitric acid. In the course of eight or ten days the patient is usually so far well that he may eat and drink what he pleases, in moderation, and also take exercise in the open air. In a few days more his recovery is complete.

Among the cases to which I have alluded in this lecture, several possessed peculiar interest, in so far as regards the views which I have now endeavored to explain. One of the unsuccessful examples, according to Roux's plan, occurred to our esteemed friend, Mr. Bowman. I saw the operation performed; it was admirably executed, and every thing as regarded the favorable condition of the parts justified a sanguine hope of success. Yet in eight-and-forty hours all the stitches had given way, and the gap was as open as ever. Some years afterwards Mr. Bowman repeated the operation, with the addition of the incisions recommended by me, and a few weeks afterwards I saw the patient with a palate as entire as if it had been so from birth. One of my own cases was still more remarkable. J. T. had been operated on by Mr. Tuson, of the Middlesex Hospital, three different times, according to the method of Roux, and the opening was left in all probability larger than it was at first. There had been a small point of union about the middle of the gap, which by the traction on the flaps had been stretched out into a narrow band, not thicker than a bit of twine. Mr. Tuson was polite enough to send this patient to me: I operated, and had the satisfaction of securing union throughout the greater portion of the soft palate. When it is considered that the edges of the fissure had been three times pared before I myself touched them, it must be admitted that the parts were in a far less satisfactory condition for an operation than at first.

Besides the instance above referred to, as occurring to Mr. Bowman, Mr. Partridge has had a successful one, and so has M. Simon; in both of these, however, some secondary proceedings were requisite. One of the most successful examples of my own proceeding occurred to Mr. Storks. Union took place throughout, and in the course of a fortnight, it would have been difficult to discover what had been the state of the palate before, or what had been done by the surgeon.

Even under the most favorable circumstances, a portion of the gap may not unite, or may open again in the course of a day or two, and it is then requisite to repeat part of the operation; the edges must be pared afresh, and one or more stitches passed, as may be necessary. This should not be done till after the lapse of weeks or months. An opening left after the first operation, though of such a size as to permit the air to pass readily through it for ten or twenty days, may, after some little further time, so close as to cause little more annoyance.

In infancy, the principal evil from cleft palate is, that the food is apt to escape upwards into the nostrils, but as the child grows older he acquires such use of the parts that such an occurrence rarely happens. The voice now appears as the principal defect, and it is not difficult for one familiar with such cases to foretel the state of the part as soon as he hears the person speak. If there be harelip, or the cicatrix resulting from an operation, for this defect suspicion may be so much the stronger, but of course no one can say with accuracy, what is the state of the palate until it is thoroughly inspected. As the chief cause of annoyance is from the voice, the principal object of the operation is to effect a favorable change upon it. There has, perhaps, been less said or written on this subject than it demands. The surgeon has usually been content with giving an account of the condition of the palate after an operation, but it is as interesting to know something of the voice. On this subject a variety of statements have been made in accordance with the experience derived from isolated cases. Sometimes the effect seems almost beyond belief, so great is the improvement; but in other instances there is no perceptible amendment. This latter result often arises from an aperture in the hard palate, with which the surgeon may not have meddled, and, until it is closed with some sort of obturator, the air escapes so freely through it that the benefit from closing the gap in the soft parts cannot be appreciated to its full extent. Even in such a case, however, the tone of the voice will probably have been greatly altered for the better; and if the opening alluded to be filled up, it may sound almost natural. It would yet be foolish to imagine that speech should be perfect, for how could this be with a person who had never articulated distinctly at any period of life? Some have been so sanguine as to expect this, and have been greatly disappointed. The fact is, that the party, however successful the operation may have been, has still to learn both how to modulate the voice, and to articulate. Some make great progress, and in the course of a few weeks or months the result is such as to please the most fastidious. In others the changes are more slow, for a year or more may elapse before much improvement can be noticed; and there are some who from want of ear, of power, disposition or perseverance, never make any satisfactory progress. In some of my own cases there has been all the improvement that could reasonably have been anticipated, but in others there has been very little. This I have attributed in some to the remaining imperfections in the palate, and in others to the want of care on the part of the patient to improve the pronunciation. I believe that all persons with this malformation have it in their power to speak more distinctly than they generally do. In one of the worst cases of cleft palate which I have ever seen, the party could articulate with considerable accuracy, and this was attributable to the care which she had bestowed in improving her voice. If, after an operation is successfully performed, the individual sets earnestly and methodically about modulating the voice and articulating distinctly, there seems nothing to prevent both being brought to a natural and average perfection. One of my patients was so zealous in his after studies that he soon spoke with more distinctness and accuracy than is generally observed in persons in whom the palate has been originally well formed. I have known some, however, so stupid, obstinate, or careless, that they could not, or would not, pronounce the word "yes," excepting in the old way. A person with cleft palate seldom sounds the "s" at the end of the word, because the air in expiration passes mostly through the fissure and nostrils. Even after the operation, the air is likely to pass by the nostrils, unless the person be careful to open the lips properly. If he does this and pushes the tongue forward with its tip against the lower front teeth, the "s" will then become distinct; and if this little lesson be readily undertaken and ably performed, there may be good hopes of speedy and great future improvement.

